# Supplementary material for: Non-invasive adapted N-95 mask sampling captures variation in viral particles expelled by COVID-19 patients: Implications in understanding SARS-CoV2 transmission
Source: PLoS One. 2021 Apr 12;16(4):e0249525. doi: 10.1371/journal.pone.0249525 (PMC8041197; doi:10.1371/journal.pone.0249525)
Supplement: S1 File — (DOCX) [file pone.0249525.s001.docx]

**Non-invasive adapted N-95 mask sampling captures variation in viral particles expelled by COVID-19 patients: Implications in understanding SARS-CoV2 transmission**

**Supplementary Information**

**Mask Sampling**


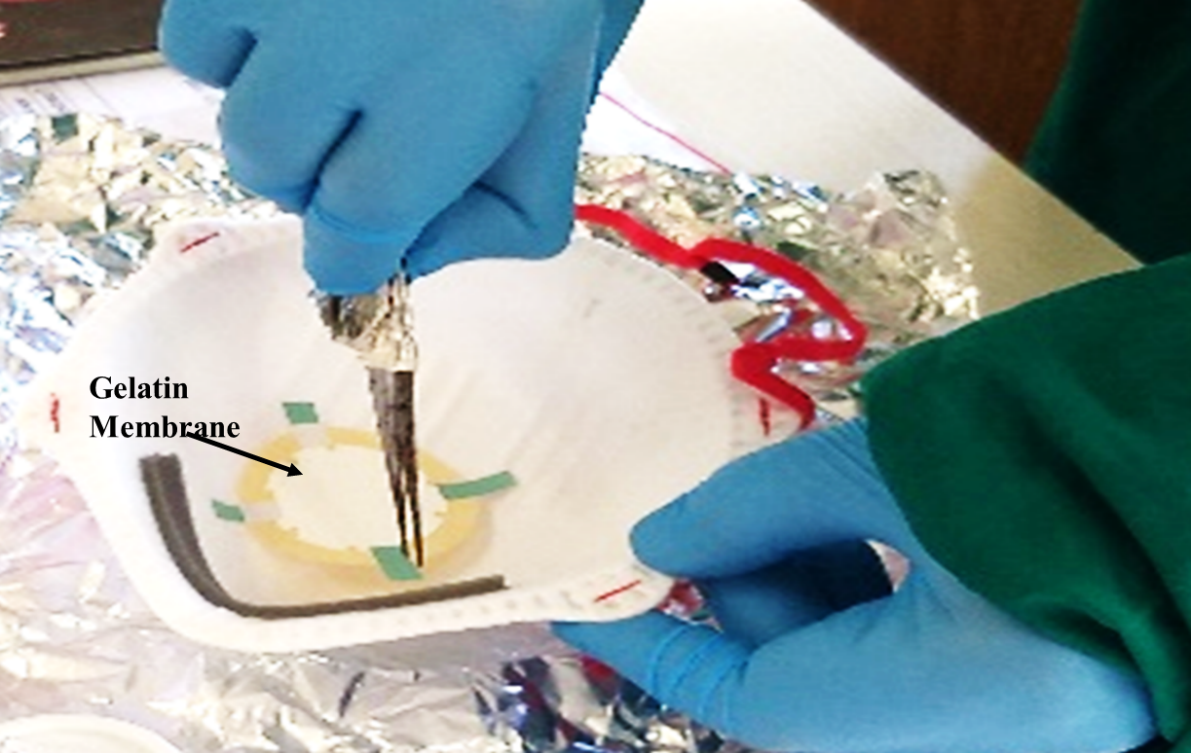


**Fig S1: N95 mask lined with gelatin membrane**: The picture depicts the removal of gelatin membrane from the mask using sterile forceps after completion of mask sampling

**Fig S2: Standard Curve for Ct values of the *E* Gene vs. Log 10 of viral copy numbers**. A positive correlation (R^2^=0.9775) between Ct values estimated for *E* gene and corresponding viral copy number in a ten-fold serial dilution of SARS-CoV-2 *E* gene-positive control provided as part of altona Diagnostics IVT kit. Equation Y = -3.264*X + 40.64 generated from the standard curve multiplied by the elution volume was used to interpolate the viral copy numbers expelled by the mask positive patient in 30 minutes.

**Questionnaire for assessing sampling score:**

The format of the case record form questionnaire used by the sample collector to note the intensities of the vocal task performed is as given below:

1. **Participant compliance information and experience with mask sampling: (Please tick the appropriate option)**
   1. While sampling,
      1. Task1: Talked/Read/Sang/recited prayer/Recited poem
         1. Volume of Task 1: Loud/Normal/Low
      2. Task 2 Coughing: Intermittent/Continuous
         1. Task 2 Coughing Intensity: Light/Deep and forceful
      3. Task 3 Breathing:
         1. Shallow/ Deep
   2. Post Sampling, participant felt easier and comfortable with

Mask sampling/Swab Sampling

**Estimation of Sampling Quality through Ct levels of RnaseP**

The human RnaseP gene's Ct values in the mask sample were used as an indicator of sampling quality. The absence of the RnaseP Ct value was considered as inadequate or inappropriate sampling. We have retrospectively analyzed the RnaseP Ct value of each mask sample using the TaqPath SARS-CoV-2 V1 detection kit (Thermo Fischer Scientific) as per manufactures protocol. We observed detectable RnaseP Ct values in all the mask samples (Median Ct value 27.29), confirming that mask sampling was done adequately. Additionally, as shown in Figure S3, we plotted the Ct values of RnaseP with the Ct value of the E gene or sampling score from all the patient samples. We found no correlation in either of the comparisons.


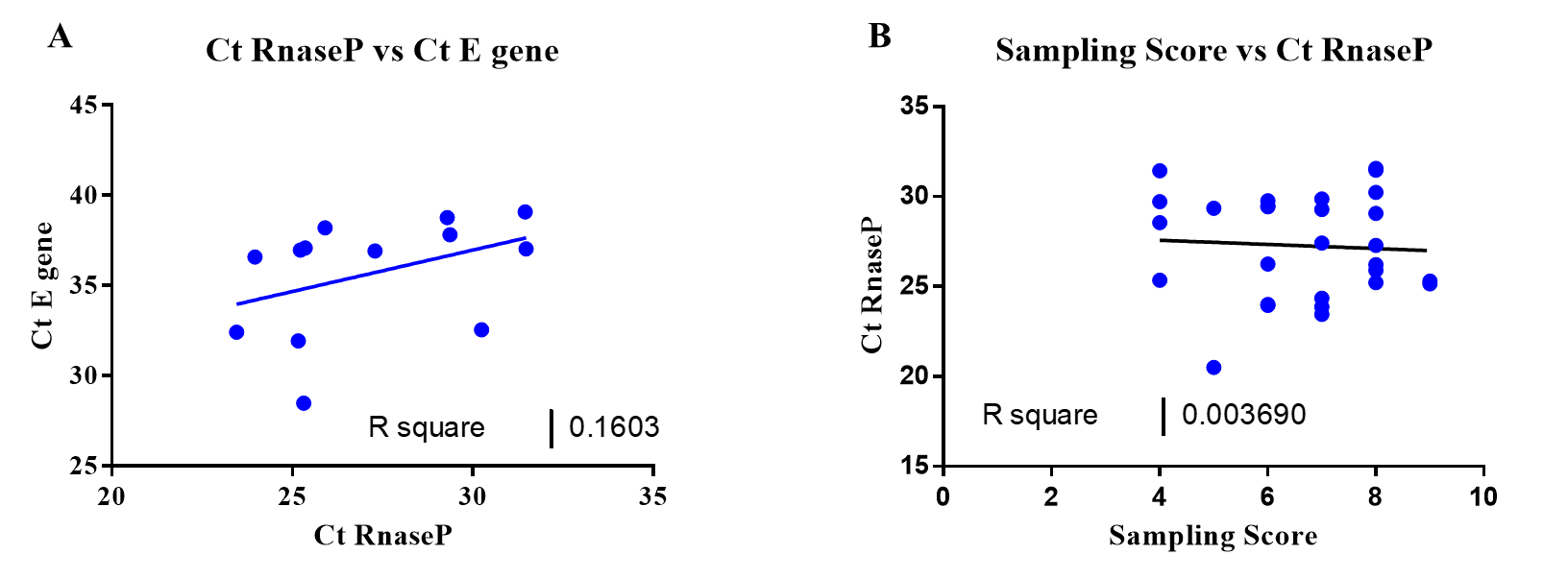


**Fig S3: Ct of RnaseP vs. Ct of E gene and Sampling Score**: Scatter plot depicting (A) RnaseP Ct values vs. E gene Ct values and (B) RnaseP Ct values and sampling score. No correlation was observed between Ct values for RnaseP and mask Ct values for E gene (R^2^ = 0.1603) or sampling score (R^2^ = 0.0036). These plots suggest that the viral output was independent of the amount of total RNA isolated from each patient sample.

**Fig S4: Ct of E gene in mask samples vs. Sampling Score:** Scatter plot depicting Sampling score for corresponding Ct of E gene. The thick black line demarcates the Mask positive samples from the mask negative samples. The mask negative samples have no detected Ct values; thus, a Ct value of 45 (last cycle of RT-PCR) has been used to depict these samples.

**Table S1: Comparison of viral load, NPS Ct, symptoms, treatment, and mask sampling characteristics among mask positive patients**

|  | Mask Positive (n=13) | |  |
| --- | --- | --- | --- |
| Descriptions | Low Emitters | High Emitters | p-value |
| Number | 9 (69.2) | 4 (30.7) |  |
| Patient Characteristics |  |  |  |
| Gender |  |  |  |
| Male | 7 (77.7) | 4 (100) |  |
| Female | 2 (22.2) | 0.0 |  |
| Age, Years |  |  |  |
| Age Median (IQR) | 44 (39.5-53) | 41 (37.2-61.2) | 0.566 |
| 20-40 | 2 (22.2) | 1 (25) |  |
| 41-60 | 7 (77.7) | 2 (50) |  |
| >60 | 0.0 | 1 (25) |  |
| Comorbidities (Diabetes/Hypertension) | 3 (33.3) | 2 (50) | 1 |
| COVID-19 Characteristics |  |  |  |
| Viral Load (Viral copies expelled in 30 minutes) | 52.89 (27.80-74.21) | 2269 (1421-16411) | **0.0028** |
| Antigen positivity at diagnosis | 6 (66.7) | 4 (100) |  |
| Median (IQR) NPS Ct of N gene if rRT-PCR+ at Sampling | 28 (22-30) | 23 (15.2-25.5) | 0.566 |
| Contact History |  |  |  |
| No Known Contact | 5 (55.5) | 1 (25) | 0.559 |
| Known contact (Family member or colleague) | 4 (44.4) | 3 (75) |  |
| Symptoms |  |  |  |
| Median (IQR) Duration of Symptoms | 6 (3-8) | 3 (2.6-4.5) | 0.088 |
| Sore throat | 4 (44.4) | 2 (50) | 1 |
| Fever | 9 (100) | 4 (100) | 1 |
| Cough | 7 (77.8) | 3 (75) | 1 |
| Breathing difficulty | 6 (66.7) | 0.0 | 0.069 |
| Loss of Smell/Taste | 5 (55.6) | 2 (50) | 1 |
| GI symptoms (Loose stools, Nausea) | 3 (33.3) | 0.0 | 0.496 |
| Weakness/Body ache/Headache | 3 (33.3) | 1 (25) | 1 |
| Median (IQR) number of symptoms | 4 (3.5-5.5) | 3.5 (1.5-4) | 0.165 |
| COVID-19 Disease Status |  |  |  |
| Mild | 6 (66.7) | 2 (50) | 0.5686 |
| Moderate | 3 (33.3) | 2 (50) |  |
| Drugs |  |  |  |
| Doxycycline | 5 (55.6) | 3 (75) | 0.506 |
| Ivermectin | 5 (55.6) | 3 (75) | 0.506 |
| Azithromycin | 0 | 1 (25) | 0.118 |
| Favipiravir | 4 (44.4) | 1 (25) | 0.506 |
| Cephalosporin | 9 (100) | 3 (75) | 0.118 |
| Hydroxychloroquine | 2 (22.2) | 0 | 0.305 |
| Sampling |  |  |  |
| Sampling Score median | 7 (4.5-8) | 8.5 (7.2-9) | 0.089 |
| Sampling Preference |  |  |  |
| Only Mask | 7 (77.8) | 3 (75) |  |
| Both Mask and Nasopharyngeal Swab | 0 | 0 |  |
| Only Nasopharyngeal Swab | 1 (11.1) | 1 (25) |  |
| Neither Mask nor Nasopharyngeal Swab | 1 (11.1) | 0 |  |

Data here represents no. (%) of subjects, unless otherwise indicated.

Abbreviations: NPS- Nasopharyngeal Swab, IQR- Interquartile range, Ct- Cycle Threshold, rRT-PCR- Real-time reverse transcriptase polymerase chain reaction

p-value Low Emitters vs. High Emitters; p-value significant at p<0.05-highlighted in bold
